# Fundoscopic Changes in Maroteaux-Lamy Syndrome

**DOI:** 10.1155/2019/4692859

**Published:** 2019-12-24

**Authors:** Augusto Magalhães, Jorge Meira, Ana Maria Cunha, Raul Jorge Moreira, Elisa Leão-Teles, Manuel Falcão, Jorge Breda, Fernando Falcão-Reis

**Affiliations:** ^1^Departament of Ophthalmology, Centro Hospitalar Universitário de São João, Porto, Portugal; ^2^Reference Centre of Inherited Metabolic Diseases, Centro Hospitalar Universitário de São João, Porto, Portugal; ^3^Department of Surgery and Physiology, Faculty of Medicine of University of Porto, Porto, Portugal

## Abstract

**Purpose:**

To describe a clinical case of mucopolysaccharidosis type VI (MPS VI), or Maroteaux-Lamy syndrome, with fundoscopic alterations that may correspond to scleral deposits of glycosaminoglycans.

**Materials and Methods:**

Clinical case report.

**Results:**

A 16-year-old girl with MPS VI was examined at the Ophthalmology Department for poor vision due to opacified corneas. Treatment consisted of bilateral penetrating keratoplasty. Retinographies and enhanced depth imaging optical coherence tomography (EDI-OCT) were performed after surgery, suggesting the presence of scleral glycosaminoglycan deposits. The patient evolved with stable corneal and fundoscopic findings.

**Conclusions:**

To our knowledge, this is the first case of MPS VI described *in vivo* with suspected deposits of glycosaminoglycans in the sclera. Fundoscopic alterations are not usually included in the ocular pathological spectrum of MPS VI. However, with improved control of systemic comorbidities, survival rates of these patients have increased, which in turn has made it possible to observe other changes besides the ones that were classically described. Despite being particularly challenging to manage, efforts should be made to maximizing the visual acuity of these patients, in order to provide them the best possible quality of life.

## 1. Introduction

Mucopolysaccharidoses (MPSs) are a group of disorders caused by inherited defects in lysosomal enzymes resulting in widespread intra and extracellular accumulation of glycosaminoglycans [1].

MPS type VI (MPS VI), or Maroteaux-Lamy syndrome, is a very rare disorder with an incidence ranging from 0.36 to 1.30 per 100,000 [2]. It is an autosomal recessive disorder caused by a deficient activity of the enzyme *N*-acetylgalactosamine 4-sulfatase, which is involved in the degradation of the glycosaminoglycans dermatan sulfate and chondroitin 4-sulfate. Deficient levels of this enzyme lead to the accumulation of partially degraded glycosaminoglycans in tissues and organs, which in turn causes a wide range of clinical manifestations, including abnormal structural development, lung infections, sleep apnoea, cardiac valvular disease, and a characteristic facies, with enlarged tongue, flat nasal bridge, and macrocephaly, that progressively worsens with age. Affected patients are usually said to be intellectually normal [2, 3].

Ocular accumulation of glycosaminoglycans results in progressive corneal opacification and is the main reason for the low visual acuity of these patients as well as the difficulty in observing the retina and optic nerve in detail. Ocular hypertension and glaucoma are also characteristic [4]. Retinal changes are not usually associated with MPS VI [3–5].

## 2. Case Presentation

A 16-year-old caucasian female with biochemical and genetic diagnosis of MPS VI (urinary glycosaminoglycans and decreased arylsulfatase B activity and c.944G> A; pR315Q homozygous mutation in the arylsulfatase B gene) was observed at the Paediatric Ophthalmology clinic with bilateral progressive vision loss in recent years.

The patient had short stature and coarse facial features, typical of MPS disease. Structural abnormalities of the upper respiratory tract had resulted in tracheostomy. Other findings included mild hearing loss, hepatosplenomegaly, cardiac valvular disease, and changes in musculoskeletal development. There was no relevant family history. She was on enzyme replacement therapy with galsufatase 1 mg/kg per week, since the age of ten.

**Figure 1:**
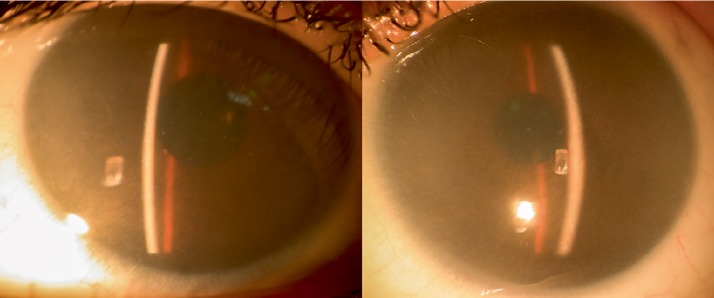
Baseline anterior segment photographs. Both corneas are cloudy due to stromal deposits of glycosaminoglycans.

**Figure 2:**
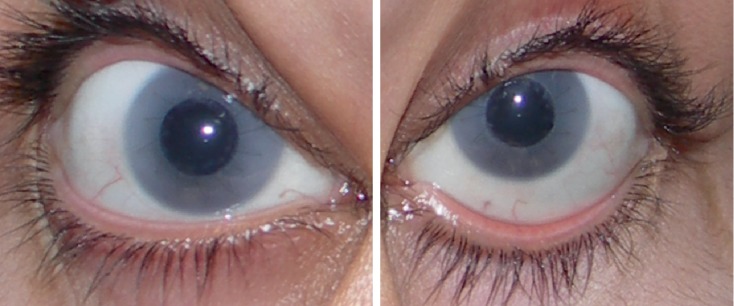
Anterior segment photographs after penetrating keratoplasty. There is central transparency of the graft with marked opacity of the peripheral host cornea.

**Figure 3:**
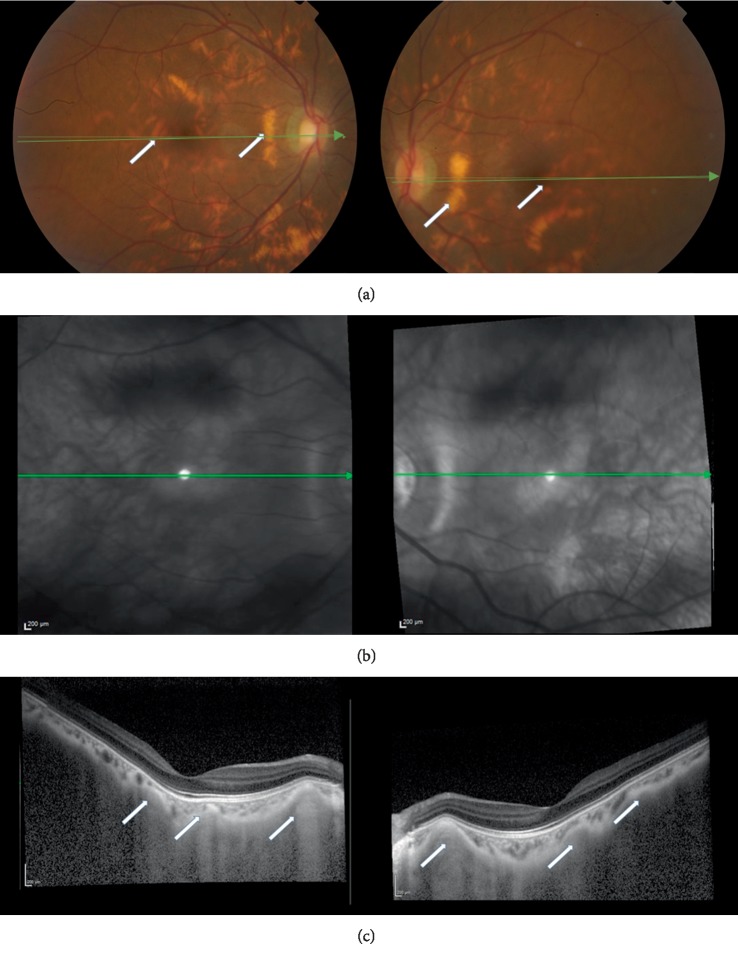
(a) Retinographies after penetrating keratoplasty. Multiple areas of orange patches are observed on retinography (arrows). (b) Infrared imaging of the fundus. (c) Enhanced depth imaging optical coherence tomography—the orange patches observed in the retinographies correspond to areas in which deposits of intermediate reflectivity can been seen at the scleral level (white arrows). These lesions are associated with marked choroidal thinning.

She presented with a best corrected visual acuity (BCVA) of 4/10 (−2.25 × 180) bilaterally. Both corneas were opacified (Figure 1), with increased thickness (central corneal thickness of 741 *µ*m and 779 *µ*m) and intraocular pressure of 34 mmHg and 32 mmHg in the right and left eye, respectively (Goldmann applanation tonometry). She was started on dorzolamide 20 mg/ml + timolol 5 mg/ml. Her corneal opacifications prevented clear ocular fundus evaluation. As corneal opacity progressed, visual acuity decreased to 3/10 in right eye and 1/10 in left eye after two years of follow-up. The patient was submitted to bilateral central penetrating keratoplasty. The trepanation diameter of the donor was 6.50 mm and the trepanation diameter of the recipient cornea was 6.00 mm. (Figure 2). After transplantation, fundoscopic evaluation became possible. Color fundus photography and enhanced depth imaging optical coherence tomography (EDI-OCT) (Figure 3) were performed. Color fundus photography revealed multiple orange patches in the macular area and around the temporal retinal vessels with no specific pattern. However, the retinal periphery was normal. The optic nerves showed a slight symmetrical pallor. EDI-OCT imaging at the location of the orange patches (Figures 3(a) and 3(b)), revealed scleral thickening associated with choroidal thinning (Figure 3(c)). The retinal pigment epithelium and retina had no apparent changes even in the areas in which the choroidal thinning and scleral thickening were evident. After 8 years of follow-up, the patient's grafts remain transparent with a BCVA of 5/10. IOP has been controlled with a combination of dorzolamide 20 mg/ml and timolol 5 mg/ml (18 mmHg in both eyes).

## 3. Conclusion

The most frequently described ocular manifestations of MPS VI are corneal clouding and ocular hypertension. Retinopathy is not frequently described amongst the usual MPS VI manifestations [1–5]. Poor vision is mainly explained by the corneal changes.

Recently, Lin et al. described retinal pigment epithelium changes in half of the patients with MPS VI [6]. However, the authors do not describe specific fundus characteristics for MPS VI patients, nor do they relate alterations to the OCT changes, that we observed. We hypothesize that scleral thickening and underlying choroidal thinning may result from scleral deposits of glycosaminoglycans. Even though we cannot be sure of the origin of the scleral thinning before a post-mortem analysis, Kenyon et al. in 1972, has described the presence of scleral glycosaminoglycan deposits in post-mortem histology cases [7].

The absence of similar case reports in the literature may be due to several reasons. Firstly, these scleral deposits may indeed be uncommon. Secondly, even if present, these deposits are not easily noticed because most patients have severe corneal opacities. Finally, the improved control of systemic comorbidities in the last years increased the life expectancy in these patients, allowing for both the development of new clinical manifestations and a better evaluation of previously unknown characteristics. It is possible that these scleral deposits may be a late manifestation in the course of the disease.

Our patient's retinal and choroid findings have remained stable over the last 2 years; however long-term prognosis of these lesions is still unknown. Due to their external location, they probably will not impair visual function. However, careful follow-up should be advocated in order to detect further changes.
